# Long-Term Outcomes of Dose-Escalated Hypofractionated Radiotherapy in Localized Prostate Cancer

**DOI:** 10.3390/biology11030435

**Published:** 2022-03-11

**Authors:** Antonio Lazo, Alejandro de la Torre-Luque, Gregorio Arregui, Daniel Rivas, Ana Serradilla, Joaquin Gómez, Francisca Jurado, María Isabel Núñez, Escarlata López

**Affiliations:** 1 Department of Radiation Oncology, San Cecilio Clinical University Hospital, 18016 Granada, Spain; antonio.lazo.sspa@juntadeandalucia.es; 2 Department of Legal Medicine, Psychiatry and Pathology, CIBERSAM, Complutense University of Madrid, 28040 Madrid, Spain; 3 Department of Physics, GenesisCare, 18004 Granada, Spain; gregorio.arregui@genesiscare.es; 4 Department of Radiation Oncology, GenesisCare, 29018 Malaga, Spain; daniel.rivas@genesiscare.es; 5 Department of Radiation Oncology, GenesisCare, 18004 Granada, Spain; ana.serradilla@genesiscare.es; 6 Department of Radiation Oncology, Torrecardenas Hospitalary Complex, 04009 Almeria, Spain; joaquin.gomez.oliveros.sspa@juntadeandalucia.es; 7 Department of Radiation Oncology, GenesisCare, 14012 Cordoba, Spain; francisca.jurado@genesiscare.es; 8 Department of Radiology and Physical Medicine, Granada University, 18012 Granada, Spain; 9 Biopathology and Regenerative Medicine Institute (IBIMER), Centre for Biomedical Research, Granada University, 18016 Granada, Spain; 10 Biosanitary Research Institute, ibs. Granada, 18012 Granada, Spain; 11 Department of Radiation Oncology, GenesisCare, 28043 Madrid, Spain; escarlata.lopez@genesiscare.es

**Keywords:** dose-escalation, hypofractionated radiotherapy, prostate cancer, volume modulated arc therapy (VMAT), Expanded Prostate Cancer Index Composite (EPIC)

## Abstract

**Simple Summary:**

Moderately hypofractionated radiotherapy (HFRT) has been shown to be isoeffective compared to conventional regimens in the treatment of prostate cancer (PCa). In addition, dose-escalation improves biochemical or metastasis control with minimal toxicity, although no overall survival benefit has been demonstrated. This work reports the results of HFRT on biochemical control, overall survival, toxicity and quality of life in patients with localized PCa treated with a dose-escalated schedule (66 Gy, 3 Gy/fraction) using volume modulated arc therapy (VMAT).

**Abstract:**

This retrospective study aimed to provide some clinical outcomes regarding effectiveness, toxicity, and quality of life in PCa patients treated with dose-escalated moderately hypofractionated radiation therapy (HFRT). Patients received HFRT to a total dose of 66 Gy in 22 fractions (3 Gy/fraction) delivered via volume modulated arc therapy (VMAT) in 2011–2016. Treatment effectiveness was measured by the biochemical failure-free survival rate. Toxicity was assessed according to the criteria of the Radiation Therapy Oncology Group (RTOG) and quality of life according to the criteria of the European Organization for Research and Treatment of Cancer (EORTC). In this regard, quality of life (QoL) was measured longitudinally, at a median of 2 and 5 years after RT. Enrolled patients had low-risk (40.2%), intermediate-risk (47.5%), and high-risk (12.3%) PCa. Median follow-up was 75 months. The biochemical failure-free survival rate was 94.2%. The incidence of acute grade 2 or higher gastrointestinal (GI) and genitourinary (GU) toxicity was 9.84% and 28.69%, respectively. The incidence rate of late grade 2 or higher GI and GU toxicity was 1.64% and 4.10%, respectively. Expanded Prostate Cancer Index Composite (EPIC) scores showed that the majority of patients maintained their QoL. HFRT to 66 Gy with VMAT was associated with adequate biochemical control, low toxicity and good reported GU and GI quality of life.

## 1. Introduction

The incidence rate of prostate cancer (PCa) is rapidly increasing, and it has become the most prevalent solid tumor diagnosed in men [1]. Radiation therapy (RT) is the gold standard alternative to surgery for localized PCa. Over the last decade, there have been three major advances in the use of external RT for the management of clinically localized PCa: (a) androgen deprivation therapy (ADT) [2]; (b) image-guided RT (IGRT) with three-dimensional conformal radiotherapy (3DCRT) and intensity modulated radiotherapy (IMRT) [3,4], and particularly c) radiation dose escalation schedules compared with conventional 70–72 Gy (2 Gy per fraction), which has been shown to improve biochemical and distant metastases control with minimum toxicity, but not overall survival [5,6,7,8,9]. In addition, novel RT techniques such as volume modulated arc therapy (VMAT) minimize the dose to the tissues surrounding the tumor, reducing the overall treatment time (OTT) of the radiation dose escalation as VMAT shows similar highly conformed doses, coverage and homogeneity of PTV and OAR savings to other IMRT techniques. Moreover, VMAT treatment plans are more efficient with fewer monitoring units and shorter treatment delivery time [10].

On the other hand, the use of conventional fractionated radiation therapy (CFRT) with a single 1.8–2 Gy fraction/day is related to more RT sessions and longer treatments; therefore, influencing the decision-making and patient non-adherence, since longer treatments can affect their financial situation (by not being able to work during radiotherapy) or cause a disruption in their daily life [11]. Additionally, mounting evidence suggests that the α/β values for PCa might be lower than 3 Gy (even <1.5 Gy) and that might increase sensitivity to a higher dose per fraction [12,13]. Consequently, several studies have explored different HFRT schedules to reduce the number of sessions and resource consumption as well as to improve the therapeutic outcome by increasing the dose per fraction (increasing improved tumor control with low side effects) [14,15,16,17,18].

HFRT and CFRT schedules have been shown to be isoeffective in terms of incidence of biochemical control or late complications in several phase III trials [19,20,21,22,23,24,25]. Furthermore, as for maintaining safety and quality of life (QoL), hypofractionation is a convenient and promising therapeutic alternative in a pandemic context as it reduces the number of visits to medical facilities.

Despite the advances in technology and knowledge in radiobiology, the beneficial impact of regimens that combine HFRT with higher dose-escalation is unclear in terms of survival and there are still concerns regarding late toxicity or improved QoL.

The main objectives of this work are to report long-term clinical results in terms of disease control rates, toxicity outcomes, and patient reported outcomes (PROs) in a series of 122 patients with localized PCa treated with VMAT and dose-escalated HFRT (66 Gy) during a short schedule of 22 sessions. In addition, specific objectives include determining possible predictive factors for clinical response, toxicity and QoL.

Although HFRT was not the standard of care at the time of accrual, the existing literature at that moment provided a solid starting point to support the hypothesis that HFRT and dose-escalation using VMAT could provide good disease control, acceptable toxicity, and the added benefit of a shorter OTT in patients with localized Pca. This work contributes to the knowledge and clinical experience in the absence of phase III clinical trials with this radiation schedule.

## 2. Materials and Methods

### 2.1. Patients

We have retrospectively analyzed the medical records of 122 consecutive patients with localized PCa treated with HFRT delivered by VMAT from January 2011 to November 2016 in two radiotherapy centers, one in the city of Granada and the other in Cordoba (Spain). All the patients were informed and signed the written consent form. Eligible patients had histologically confirmed primary PCa and were not candidates for pelvic irradiation based on the risk of extraprostatic extension evaluated by Partin nomograms [26]. Patients who required elective pelvic irradiation were excluded, as they are treated, according to our protocols, with a regimen of 50.4 Gy in the pelvis (1.8 Gy/session) with a boost in the prostate up to 70 Gy (2.5 Gy/session). ADT was prescribed for 6 or 24 months in intermediate or high-risk PCa patients, respectively, according to the D’Amico classification system and the physician criteria in every particular case [27]. The ADT regimen combined luteinizing hormone-releasing hormone (LHRH) agonists plus antiandrogens (the first 30 days), starting two months before the beginning of radiotherapy.

Intermediate risk was defined as PSA 10–20 ng/mL, T2b/T2c or Gleason 7; and high risk as PSA > 20 ng/mL, ≥T3 or Gleason ≥ 8. Values lower than these corresponded to the lower risk group. The American Joint Committee on Cancer (AJCC) TNM system (7th edition) was used for the staging [28] and the Charlson index was used to evaluate comorbidities in PCa patients [29]. An imaging staging study with bone and CT scan was performed in intermediate and high-risk patients.

### 2.2. Radiotherapy

Radiation was delivered by VMAT supported by two linac (linear accelerators) twins Elekta Synergy MLCi2^®^ (Elekta AB, Stockholm, Sweden), one in each of the two RT centers. The treatment plan consisted of 1 or 2 arcs of 6 MV beams with a variable number of segments. For dosimetry planning, computed tomography (CT) images were obtained from the L5 vertebrae to below the ischial tuberosities with 3 mm slice thickness. Patients were placed in the supine position, with a comfortably full bladder and empty rectum. Clinical target volume (CTV) delineation included the prostate and the proximal 1 cm or 2 cm of seminal vesicles in intermediate and high risk PCa, respectively [30]. The CTV was expanded uniformly by 5 mm to create the PTV.

Patients received a total dose of 66 Gy in 22 fractions (3 Gy/fraction) delivered 5 days/week over 4.5 weeks, equivalent dose in 2 Gy fractions (EQD2) 84.9 Gy, assuming an α/β value of 1.5 Gy for PCa and 5.0 Gy for normal tissues. Dose prescription to CTV aimed to cover 100% of this volume and 95% of the PTV (D95).

**Figure 1:**
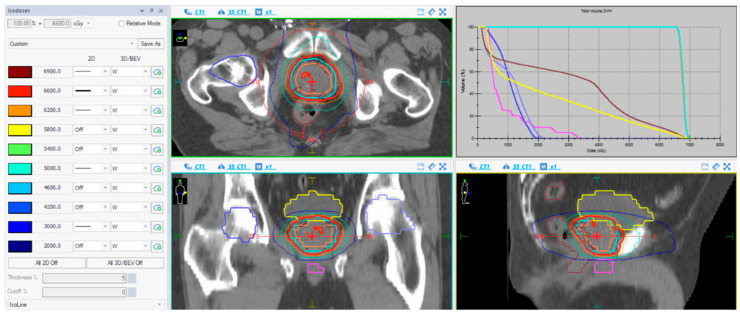
Dose-volume histogram (upper-right image); a blue line represents the PTV coverage, brown and yellow lines represent the dose received by the rectum and bladder, respectively. CT images of volumes and isodose distribution of a VMAT plan in the axial, coronal and sagittal planes (the rest of images); a blue line represents PTV margin. Treatment plan showing the radiation dose distribution in colored, lines within the interval from 30 Gy (dark blue) to 66 Gy (red).

The bladder, femoral heads, penile bulb and the whole rectum were contoured in all patients. The rectal constraints were V40 < 55%, V48 < 50%, V53 < 30%, V58 < 15% and V62 < 3%. The bladder constraints included V40 < 50%, V48 < 25% and V62 < 5%. Prior to each radiation session, cone-beam CT was used as image-guide radiation therapy (IGRT) along with a HexaPOD™ 6D Robotic Treatment CouchTOP (Elekta AB, Stockholm, Sweden) with six degrees of freedom to correct for any misalignments detected by CT, which allows a planning target volume (PTV) margin of 5 mm, which is smaller than the margin of the conventional 3DCRT approach used in radical treatments without IGRT (Figure 1).

### 2.3. Clinical and Toxicity Assessment Follow-Up

The analyzed clinical data included biochemical failure and overall survival. Biochemical relapse was determined using the Phoenix definition (PSA nadir + 2 ng/mL) [31]. Acute and late toxicity was determined according to the toxicity criteria of the Radiation Therapy Oncology Group (RTOG) and the European Organization for Research and Treatment of Cancer (EORTC) [32].

Patients were first seen two months after the end of RT and in subsequent follow-up visits every 4–6 months for three years and then annually. A PSA test and physical examination were done at each visit.

Late toxicity was defined as complications occurring at least 6 months after the end of RT.

### 2.4. Quality of Life

QoL was measured on two occasions over the follow-up period, at two years and five years after the end of RT. Patients completed the Spanish validated version of the Expanded Prostate Cancer Index Composite (EPIC-26) questionnaire whose questions are scored on a 0–100 score, with a higher score indicating better QoL [33]. We focused on the GI and GU domains.

### 2.5. Statistical Analysis

Descriptive statistics helped us to understand the main features of our sample. The mean and standard deviation were used for continuous variables, and percentages for categorical and dichotomous variables. Survival curves were plotted using the Kaplan-Meier non-parametric estimator considering the biochemical failure, all-cause mortality, and PCa-specific mortality. Cox regression was used to identify predictors of survival or relapse (age, stage, PSA before RT, Gleason score, Charlson index to quantify comorbidity, and the prostate cancer risk group). The number of months since the end of RT was used as a time variable. Binary logistic regression was used to evaluate how the previously mentioned variables could predict acute or late toxicity. For the late toxicity model, the presence of acute toxicity was also included as a weighting variable. Finally, linear regression models were applied to explain the scores on the EPIC-26 scoring applied at five years, using the same predictors. For all explanatory models, the Akaike Information Criterion (AIC) was used to evaluate whether the model with predictors explained higher proportion of the criterion variance. The odds ratio (OR) was used to estimate the loading of each predictor, with a 95% confidence interval. The Wald test was used to assess whether a predictor shows a loading significantly different than 1. All analyses were performed using R 3.0.1 software x64 (RStudio Team, Vienna, Austria)(psych, survival and polycor packages).

## 3. Results

Median age at treatment initiation was 70 years (range, 54–82) and mean PSA before RT was 9.1 ng/mL (SD = 4.27). According to the risk classification, 49 (40.2%) patients had low risk, 58 (475%) had intermediate risk and 15 (12.3%) had high risk PCa. Only 1 patient with extraprostatic disease (T3a) was included, since he did not present other high-risk factors (as occurs in most of our patients) that, according to our protocols, would make him a candidate for elective irradiation of the pelvis with a different scheme. Therefore, it was possible to include this patient in the exclusive radiotherapy to the prostate with the 66 Gy schedule. Hormone therapy was prescribed to 41.8% patients (Table 1), according to the physician criteria.

### 3.1. Clinical Outcomes

**Figure 2:**
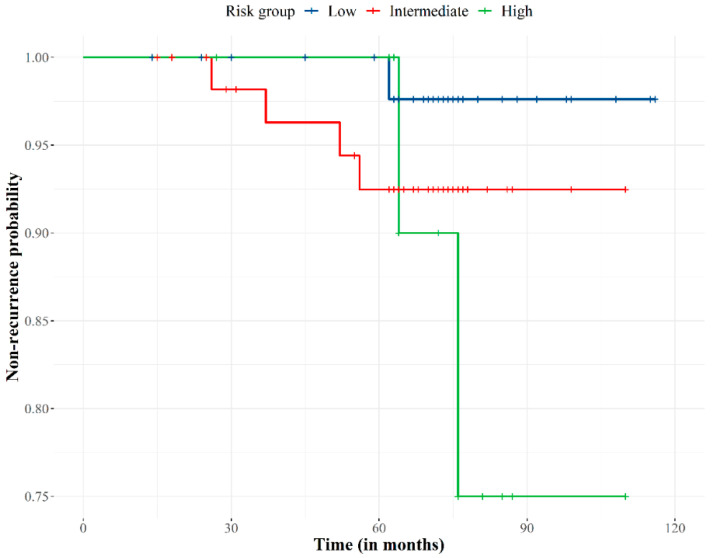
Biochemical failure-free curve according to prostate cancer recurrence risk group. Non-recurrence probability slightly decreased over 28 months of follow-up, progressively decreased to 75 months when the probability stabilized at 92%. The risk of recurrence became extreme for the high-risk group at 75 months of follow-up.

The median follow-up was 74 months (range, 15–118 months). All patients completed treatment without interruption. Eight patients were lost at the 2-year follow-up. The biochemical control rate was 94.2% (Figure 2). Seven (5.8%) out of the 122 patients developed biochemical failure, with a median time to relapse of 56 months (range, 26–65 months). Of these seven patients, three showed clinical failure at 51, 56, and 65 months. Two patients had bone and lymph node metastases, and one patient had local relapse that was treated by high dose-rate brachytherapy and who was biochemically controlled at the time.

Regarding the predictors that explain biochemical recurrence (Table 2), the PSA levels before RT and the risk group showed a significant explanatory loading (*p* < 0.01). There was an increased risk of biochemical failure in the high-risk group (in comparison to the low-risk). The rest of the predictors (age, stage, Gleason and Charlson scores) were proven to be non-significant in this analysis.

**Figure 3:**
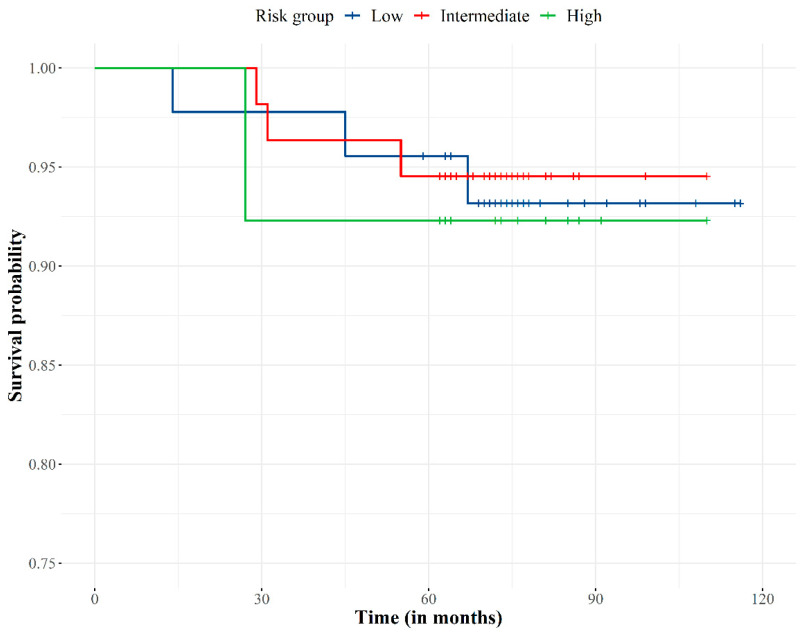
Overall survival curve according to the risk group.

The overall survival was 93.8% (Figure 3). No patients died from PCa (PCa specific survival 100%) as PSA levels and clinical data (physical examination and complementary diagnostic tests) showed patients were free of disease at time of death.

According to the multivariate regression analysis, no significant predictors were found (*p* > 0.05) for overall survival.

### 3.2. Toxicity

Treatment was well tolerated with 68% and 29% of patients not showing any symptoms of acute GI or GU toxicity, respectively. Regarding the patients that had acute GU symptoms, 41.8% of them had grade 1 toxicity, and 28.7% grade 2 toxicity. As for patients with acute GI symptoms, 22% of patients had grade 1 toxicity and 9.8% had grade 2 toxicity. No grade 3 acute toxicity was reported.

Grade 3 GU late toxicity was rare, with only one observed incontinence event (maximum value during the observation period). No patients had grade ≥3 GI toxicity. At the last follow-up, 91.8% and 78.7% of patients did not have any late GI and GU toxicities, respectively. The most common symptoms were diarrhea and tenesmus in the GI sphere, dysuria and increased frequency in the GU spectrum.

Table 3 displays the proportion of cases showing each degree of toxicity according to the risk group. Significant differences were observed between the groups only in terms of acute GI symptoms, χ^2^ (4) = 11.66, *p* < 0.05. Both the intermediate- and high-risk groups were featured by higher probability to show more severe (i.e., grade 2) acute GI toxicity.

In the regression analysis, no covariates explained acute toxicity. On the other hand, two covariates did explain late toxicity: age (OR = 1.94, CI_95_ = (1.05, 3.88), Z = 2.01, *p* < 0.05) and the PSA level before the treatment (OR = 1.77, CI_95_ = (1.07, 3.21), Z = 2.06, *p* < 0.05), observing a higher risk of late toxicity with higher age and higher PSA before the treatment.

### 3.3. Patient-Reported Outcomes

The longitudinal evaluation of the GU and GI QoL revealed that patients reported high levels of QoL in terms of urinary and gastrointestinal function (Table 4). Most of them reported no/small problems.

The comparisons between the evaluations at 2 and 5 years only found a slight decrease in QoL related to the incontinence domain, with significant differences between the incontinence assessed at 2 years and at 5 years, [*t* (102) = 3.12, *p* < 0.05]. This was not observed when analyzing data from the irritative urinary or bowel symptoms. This QoL decrease may be explained by an increased biochemical relapse probability over time and may also be associated with the presence of late toxicity, and a more locally advanced tumor (based on the TNM clinical staging classification) (Table 5).

## 4. Discussion

This work is one of the few studies that report on late toxicity and PRO results with HFRT dose escalation to 66 Gy (3 Gy/fraction), five days/week, and the only one that used VMAT. This study provides some insights into the long-term outcomes of a series of 122 patients with localized PCa treated with favorable results in terms of biochemical control, late toxicity, and PROs.

As evidence suggests, low α/β values for PCa are related to high fractionation sensitivity [12,13]; consequently, several phase III trials have explored different HFRT schedules (Table 6). Non-inferiority studies (studies designed to demonstrate the non-inferiority of new hypofractionated regimens over standard fractionated schemes) have shown that HFRT can be as isoeffective as CFRT, with no significant differences in the incidence of late complications or biochemical control rates [18,19,20].

Previous studies have shown that tumor relapse after RT mainly occurs at the site of the primary lesion [37,38]. Dose escalation may be decisive for the control of biochemical relapse, clinical failure and PCa-specific mortality, but it does not seem to improve overall survival [8]. However, so-called HFRT “superiority” trials (studies designed to demonstrate the superiority of new hypofractionated schedules), have not shown substantially better results than other HFRT schedules [24,35]. De Vries et al. found significant improvement in local control in patients with a Gleason score = 8 treated with HFRT versus conventional schedule, but not in relapse-free survival [35]. Moreover, Avkshtol et al. (Fox Chase) reported higher incidence of distant metastases at 10-year follow-up in patients in the HFRT arm (rate difference, 7.8%; 95% CI, 0.7% to 15.1%) and observed no between-group difference in overall survival [36]. Finally, the MD Anderson trial showed a 10.7% biochemical failure incidence in men treated with HFRT versus a 23.7% of incidence in patients treated with CFRT over a 10-year follow-up [25].

Regarding toxicity, HFRT has shown similar outcomes to CFRT in terms of acute toxicity. Nevertheless, there always has been a concern about possible late adverse effects linked to HFRT. Several studies have shown statistically similar late toxicity rates between the two fractionation schedules [39,40]. However, Alluwini et al., who used the highest EQD2 (90.4 Gy), reported an increased cumulative incidence of late GI toxicity (stool frequency) grade ≥ 2 (1.19 Hazard Ratio (HR) [95% CI 0.93–1.52]) and an increased cumulative incidence of late GU toxicity (frequency, nocturia and incontinence) grade ≥ 3 with HFRT versus CFRT (19.0% [95% CI 15.2–23.2] vs. 12.9% [9.7–16.7], respectively; *p* = 0.021) [22]. Pollack et al. found no differences in late toxicity, but in subgroup analysis found that patients with urinary dysfunction prior to enrollment showed worsening urinary symptoms after HFRT [23].

The lack of statistically significant results from superiority trials suggests that there might be a limit to escalate the radiation dose. This adds to the doubts about the α/β ratio being lower than it had been previously thought due to the influence of a time factor. This would make the α/β value of PCa similar to the α/β value of the surrounding organs at risk [41]. In this regard, the HYPRO trial used a dose of 90.4 Gy, but failed to improve the biochemical free survival, which could be related to the longer duration of the treatment [34].

The VMAT technique is known for its less acute toxicity with an equivalent local control compared to other techniques [42,43], while improving and facilitating the dose increase with a lower number of sessions [10]. This is related to a more comfortable and shorter total treatment duration, which could be particularly convenient for patients and health providers, in a pandemic context.

Several studies have reported the outcomes of a 66 Gy scheme (3 Gy/fraction) with excellent biochemical control rates with moderate toxicity [44,45,46]. Patel et al. treated 129 patients and found an 8-year biochemical control rate of 92% with good tolerance. In this prospective observational study, no grade > 3 toxicity was observed in its cohort, and grade 2 toxicity was 27% and 33% for GI and GU, respectively. At the last follow-up, only 1.5% of patients showed any GI or GU toxicities. However, Patel et al. used 3DCRT, therefore their results cannot be extrapolated to more modern techniques [44] as 3DCRT requires wider PTV margins and lower conformation of dose distribution.

Lieng et al. conducted the only phase II trial that used 60 Gy and 66 Gy schemes. However, the study had to stop the accrual in the 66 Gy group with 33 patients, as they found that > 10% patients showed late grade 3–4 toxicity (a prespecified rule for stopping the trial). The biochemical failure free survival was 80% at eight years [45]. However, the authors used IMRT and a 10 mm–7 mm PTV margin, which is larger than the margin used in our study.

Finally, the retrospective study by Hashimoto et al. is more similar to ours. They treated 195 patients with IMRT with a median follow-up of 40 months and they reported only 6.7% biochemical failure. Acute GU toxicity grade 2 was 34.9%. No acute GI toxicity grade ≥ 2 was reported, and no late grade ≥3 was observed with a 34.9% and 2.6% of grade 2 late GU and GI toxicity, respectively. Nevertheless, the OTT (almost 7.5 weeks) was longer than the other studies because Hashimoto et al. used a three days/week regimen [46]. This schedule might have contributed to the better toxicity outcomes.

With a median follow-up of 74 months, the present study shows a biochemical free survival and overall survival of 94.2% and 93.86%, respectively, with no PCa-related deaths. These clinical results are comparable to other works using dose escalated HFRT [22,23]. In this regard, our findings could be related to the high dose delivered and to the effect of a shorter OTT (4.5 weeks in 5 days/week schedule), which may have contributed to effective local tumor control.

Biochemical free survival was found to be related to the risk group and PSA levels before RT. This may suggest that some patients (with higher PSA at the beginning of HFRT) might actually have more advanced disease than was formally established in the staging study due to presence of micrometastases.

Moderate (grade 2) acute toxicity was common in the current study, but this was not a predictor of late toxicity events. Most treated patients did not show late GU (78%) or GI (91%) toxicities. Late GI toxicity grade 3 was not observed and only one patient had late GU toxicity grade 3. As mentioned above, these outcomes are comparable in late GI toxicity and better in terms of late GU toxicity to other HFRT schedules [20,25,44,45,46]. The use of VMAT, IGRT, and a lower PTV margin (5mm) could have contributed to the low levels of toxicity related to smaller volumes of normal tissues receiving full dose. Moreover, this could also be explained because most of the patients had low to intermediate risk of localized PCa, therefore, with a smaller CTV. The difference in volumes and PTV margins may have contributed to the decreased dose in OAR and, consequently, lower toxicity. A comparative dose-volume histogram (DVH) analysis of the rectum and bladder between studies would be necessary to confirm this hypothesis in further studies.

Regarding QoL, several trials have shown favorable PROs. Despite an initial decrease in bowel domain values or urinary incontinence [47,48], statistical association has not been found between QoL deterioration and RT schedule [19,47,48,49,50]. On the other hand, QoL results of the HYPRO trial did not demonstrate the non-inferiority of its HFRT scheme. The HYPRO trial revealed a slight but statistically significant GU and GI deterioration particularly during the first 12 months [51].The study by Hashimoto et al. with a 66 Gy/3 Gy scheme is the only previous study that investigated QoL and found differences one month after RT in the GU and bowel domains, but these toxicities returned to normal status (as before RT) in 3 and 6 months, respectively [46]. Most of the patients in our study reported no to small GU or GI problems in the PRO questionnaires. The slight decline in incontinence domain was related to the presence of late toxicity and a higher initial PSA level according to the regression analysis.

Some limitations of the study are the variable and relatively short follow-up (more than 10-year data would be required to confirm these findings), the retrospective nature of the study and the absence of a control group. In this sense, Raziee et al. carried out a retrospective dose-escalation study with normofractionated schemes. A group of 355 patients, with characteristics similar to those included in our study (i.e., 12.3% high-risk patients), received 78 Gy at a rate of 2 Gy/fraction, with IMRT/VMAT and IGRT techniques [52]. With a median follow-up of 4.9 years (3.3–6.5), the study showed a biochemical control of 81.4% at 8 years, with an overall survival of 96% at 5 years (100% cancer-specific survival) with a 3.1% and 1.4% grade 3–4 late GI and GU toxicity, respectively. Although they are different studies (both retrospective) and cannot be directly compared, the results in both are favorable in terms of disease control and low late toxicity, suggesting that the 66 Gy regimen is feasible with acceptable toxicity. We also have to recognize the potential confounding related to the ADT given to intermediate to high-risk PCa patients. Compared to other studies (i.e., Devries et al.) [35], high-risk patients are barely represented, and this factor may influence the general outcomes, so they are favorable in terms of survival, as an increased risk of biochemical failure was observed in the high-risk group (but there was no difference in overall survival). Another limitation is that PROs were collected twice during the follow-up period (at 2 and 5 years after RT), but not at baseline. Furthermore, patient hormonal and sexual domains were not considered in this study, as we focused on GU and GI symptoms. Finally, the dropout rate was substantially high particularly at the second (five years) PRO assessment, which could also have had an impact on the QoL results.

Future study directions will involve moving towards the use of ultra-hypofractionated schemes, which are already showing promising results, as well as supportive techniques such as rectal spacers that may help ensure treatment safety [53,54,55]. Comparative studies with these moderate and extreme schemes and techniques are required.

## 5. Conclusions

The present study shows that HFRT to 66 Gy, 3 Gy/fraction, with VMAT is feasible and provides favorable results regarding efficacy, toxicity and QoL, by reducing OTT over other moderate hypofractionated schedules, which is more comfortable and patient convenient.

In the absence of phase III studies with this radiation schedule, this study provides information regarding a different HFRT schedule in PCa. Further comparative studies using moderate/ultrahypofractionated schedules and different technologies are recommended.

## Figures and Tables

**Table 1:** Baseline characteristics of the patients included in the study (N = 122).

Variable	Median (Range)	Percentage of Patients (%)
Follow-up (months)	75 (15–118) *	
Age (years)	70 (54–82)	
T stage		
T1c		54.1
T2a–c		45.08
T3a		0.82
ADT		41.8
Gleason score		
<7		64.7
=7		31.15
>7		4.15
Risk group		
Low		40.2
Intermediate		47.5
High		12.3
Charlson CI	3.16 (1.16)	

CI: comorbidity index; ADT: Androgen Deprivation Therapy. Note. Median range (in parentheses) are presented for continuous variables. Categorical variables are presented in percentage of cases. * The median follow-up was 74 months and included all participants. The data for survivors were (mean) = 77.31 months, standard deviation = 11.45; median = 75 months, range 15–118.

**Table 2:** Predictor coefficients that explain biochemical failure.

Predictors	OR	CI_95_ LB	CI_95_ UB	Z
Age	0.78	0.27	2.26	−0.45
Stage T (ref.: stage T1) ^a^	0.92	0.13	6.67	−0.08
PSA	28.83	4.20	198.03	3.42 *
Gleason score ^b^	0.56	0.15	2.12	−0.85
Risk group (ref.: low)				
Intermediate	6.21	0.52	74.42	1.44
High	112.84	4.71	2702.91	2.92 *
Charlson CI	0.49	0.17	1.39	1.35

Ref: reference; CI: comorbidity index; OR: Odds ratio; CI_95_ LB: Lower bound of the confidence interval. CI_95_ UB: Upper bound of confidence interval; Z: Wald’s test for the OR. Note. The Cox model with predictors had a better fit to data (AIC = 53.78) than the model without predictors (AIC = 63.49). This model with predictors did not include hormonal therapy because it had few cases without that condition. This model had 90 concordance, Wald test χ^2^ (5) = 13, *p* < 0.05. ^a^ The variable included stage T1 and T2 PCa patients. There was only one patient with stage T3 who was not included in the analysis. ^b^ This variable was dichotomized in the analysis, using the reference category Gleason < 7. * *p* < 0.01.

**Table 3:** Acute and late toxicity outcomes according to the risk group.

Toxicity	Low Risk (%)	Intermediate Risk (%)	High Risk (%)
Acute GU			
Grade 0	30.61	24.14	46.15
Grade 1	44.9	39.66	38.46
Grade 2	24.49	36.21	15.38
Acute GI			
Grade 0	69.39	63.79	76.92
Grade 1	30.61	18.97	7.69
Grade 2	0	17.24	15.38
Late GU			
Grade 0	81.63	72.42	92.31
Grade 1	12.24	24.14	7.69
Grade 2	6.12	1.72	0
Grade 3	0	1.72	0
Late GI			
Grade 0	89.8	91.38	100
Grade 1	10.2	5.17	0
Grade 2	0	3.45	0

GU: genitourinary; GI: gastrointestinal.

**Table 4:** Genitourinary and gastrointestinal EPIC domain scores at 2- and 5-year follow-up.

EPIC Domain	Mean (SD)
Incontinence *	
2 years	90.40 (19.69)
5 years	85.07 (26.58)
Irritative urinary symptoms	
2 years	92.75 (11.66)
5 years	94.18 (10.73)
Bowel symptoms	
2 years	97.38 (6.80)
5 years	96.19 (9.74)

EPIC: Expanded Prostate Cancer Index Composite; SD: standard deviation. Results are expressed as mean (standard deviation). * Significant differences between incontinence evaluated at 2 years and at 5 years, *p* < 0.05.

**Table 5:** Predictor coefficients that explain the EPIC score of the incontinence domain.

	OR	CI_95_ LB	CI_95_ UB	*t*
Age	1.00	0.96	1.03	−0.20
Clinical T stage (ref.: T1)	0.95	0.89	0.99	−1.42
PSA	1.02	0.99	1.06	1.51
Gleason score (ref.: <7)	1.02	0.95	1.10	0.63
Charlson CoI	0.99	0.96	1.03	−0.49
Biochemical failure (ref.: no)	0.87	0.77	0.99	−2.16 *
Late toxicity (ref.: grade 0)	0.87	0.81	0.94	−3.75 **
Risk group (ref.: low)				
Intermediate	0.95	0.88	1.03	−1.29
High	0.96	0.85	1.08	−0.73
	**OR**	**CI_95_ LB**	**CI_95_ UB**	***t***
Age	1.00	0.96	1.03	−0.20
Clinical T stage (ref.: T1)	0.95	0.89	0.99	−1.42
PSA	1.02	0.99	1.06	1.51
Gleason score (ref.: <7)	1.02	0.95	1.10	0.63
Charlson CoI	0.99	0.96	1.03	−0.49
Biochemical failure (ref.: no)	0.87	0.77	0.99	−2.16 *
Late toxicity (ref.: grade 0)	0.87	0.81	0.94	−3.75 **
Risk group (ref.: low)				
Intermediate	0.95	0.88	1.03	−1.29
High	0.96	0.85	1.08	−0.73

Model fit (AIC), With covariates = 875.19, Without covariates = 864.64, CoI: comorbidity index; OR: Odds ratio; CI_95_ LB: Lower margin of the confidence interval; CI_95_ UB: Upper margin of confidence interval; *t:* Wald’s test for the OR. AIC: Akaike Information Criterion. Note. The presented results are based on linear regression. * *p* < 0.05, ** *p* < 0.1.

**Table 6:** Phase III trials that have explored different HFRT schedules.

Author [Ref] (Study)	Study Design	Year	N	RT Technique	HFRT Scheme	EQD2_1.5_/EQD2_3_	Biochemical Control	Late Toxicity GU ≥ G2	Late Toxicity GI ≥ G2
Dearnaley et al. [19] (CHHiP)	Non-inferiority	2016	3216	IMRT	60 Gy/3 Gy	77 Gy/74 Gy	90.6% (5-years)	9.1%	13.7%
Catton et al. [20] (PROFIT)	Non-inferiority	2017	1206	IMRT	60 Gy/3 Gy	77 Gy/74 Gy	85% (5-years)	22%	8.9%
Lee et al. [21] (RTOG 0415)	Non-inferiority	2016	1105	3DCRT/IMRT	70 Gy/2.5 Gy	80 Gy/77 Gy	86.35 (5-years)	29.7%	22.4%
Aluwini et al. [22] Incrocci et al. [34] De Vries et al. [35] (HYPRO)	Superiority	2016	820	IMRT	64,6 Gy/3.4 Gy	90.4 Gy/82.7 Gy	80.5% (5-years)	41.3%	21.9%
Arcangeli et al. [24]	Superiority	2017	168	3DCRT	62 Gy/3.1 Gy	81 Gy/75 Gy	72% (10-years)	11%	14%
Hoffman et al. [25] (MDACC)	Superiority	2018	206	IMRT	72 Gy/2.4 Gy	80.2 Gy/77.7 Gy	89.3% (10 years)	15%	12%
Pollack et al. [23] Avkshtol et al. [36] (Fox Chase)	Superiority	2013 2020	303	IMRT	70.2 Gy/2.7 Gy	84.4 Gy/80 Gy	69.4% (10-years)	15.3%	18.1%

## Data Availability

Not applicable.
